# Developing the FIGO‐IPPS “R U MOVVING SOMe” classification system for female chronic pelvic pain

**DOI:** 10.1002/ijgo.70522

**Published:** 2025-09-08

**Authors:** Georgine Lamvu, Juan D. Villegas‐Echeverri, Catherine Allaire, Sawsan As‐Sanie, Jorge Carrillo, Susan Khalil, Andrew W. Horne, Alexander Wang, Malcolm G. Munro

**Affiliations:** ^1^ College of Medicine University of Central Florida Orlando Florida USA; ^2^ Orlando VA Healthcare System Orlando Florida USA; ^3^ ALGIA Unidad de Laparoscopia Ginecologica Avanzada y Dolor Pelvico Pereira Colombia; ^4^ Clinica Comfamiliar Pereira Colombia; ^5^ BC Women's Centre for Pelvic Pain and Endometriosis Vancouver British Columbia Canada; ^6^ Department of Obstetrics and Gynaecology University of British Columbia Vancouver British Columbia Canada; ^7^ Department of Obstetrics and Gynecology University of Michigan Ann Arbor Michigan USA; ^8^ Division of Minimally Invasive Gynecologic Surgery, Department of Obstetrics, Gynecology and Reproductive Science Mount Sinai Hospital at the Icahn School of Medicine New York New York USA; ^9^ Centre for Reproductive Health, Institute of Inflammation and Repair University of Edinburgh Edinburgh UK; ^10^ Veterans Affairs Greater Los Angeles Los Angeles California USA; ^11^ Department of Obstetrics and Gynecology David Geffen School of Medicine at the University of California Los Angeles California USA

**Keywords:** chronic pelvic pain, classification system, female, R U MOVVING SOMe

## Abstract

The goal was to develop a pragmatic classification system for conditions associated with chronic pelvic pain (CPP), aiming to enhance diagnosis, management, education, and research of CPP. An international, multidisciplinary panel participated in a modified RAND/UCLA Delphi consensus. This panel included healthcare professionals, medical society representatives, experts, individuals with lived experience of pain, advocacy groups, researchers, educators, and journal editors. The Delphi process comprised three rounds: two online surveys and one virtual meeting. Participants scored their agreement with statements using a 9‐point Likert scale (1 = strongly disagree, 9 = strongly agree). A priori criteria for consensus were defined as follows: agreement, a mean score ≥7 with <15% scoring ≤3; disagreement, a mean score ≤3 with <15% scoring ≥7. Responses not meeting these criteria were considered indeterminate and advanced for further refinement in subsequent rounds. In round 1, 65 of 77 (84.4%) stakeholders participated; round 2 consisted of responses from 54 (70.1%) stakeholders, and 34 (44.2%) stakeholders engaged in round 3. The Delphi process yielded broad consensus on the definition of CPP and a corresponding classification system with the acronym R U MOVVING SOMe. This novel system comprises 12 categories: Reproductive, Urinary, Musculoskeletal, Other (not otherwise classified), Vulvovaginal, Vascular, Idiopathic (no pain contributor identified), Neurologic, Gastrointestinal, Sensitization/Nociplastic, Overlapping pain conditions, and Mental health. The R U MOVVING SOMe classification system represents a significant step towards a standardized framework for evaluating CPP. The high level of engagement and consensus among a diverse group of international stakeholders underscores its future potential to improve communication, clinical practice, education, and research in this challenging field.

## 1 INTRODUCTION

Chronic pelvic pain (CPP) affects nearly 25% of women worldwide.^1^, ^2^ Although the definition of CPP varies slightly between medical and specialty societies, it is generally defined as pain lasting longer than 3–6 months that is associated with significant disability, multiple, often overlapping, pelvic and non‐pelvic pain comorbidities, along with pelvic floor dysfunction, including urinary, bowel, and sexual dysfunction.^3^ Research consistently shows that CPP disproportionately affects women, with a female: male ratio of 2:1.^1^, ^4^ This condition profoundly impacts quality of life, often leading to physical disability, psychological distress, and reduced social functioning.^5^ Individuals experiencing CPP frequently experience limitations in daily activities and work productivity, contributing to a substantial burden on healthcare resources and personal well‐being.^5^ Furthermore, studies frequently show that women experiencing CPP spend prolonged periods in pain and report being dismissed, marginalized, and stigmatized by their healthcare professionals and communities.^6^, ^7^

Despite its substantial impact worldwide, CPP remains a largely neglected healthcare problem.^8^ The multifactorial nature of the pain, the influence of psychological factors, lack of treatment resources, insufficient clinical training, lack of education, and clinical time constraints are often cited as some of the reasons for this neglect.^9^, ^10^ Recognizing this critical gap, FIGO and the International Pelvic Pain Society (IPPS) recently issued a consensus statement advocating for a framework designed to overcome these barriers.^8^ Critical to this framework was the first step of establishing a standardized definition and classification system for CPP that would:

- Empower patients by naming their condition, validating their experience, and facilitating communication with healthcare professionals.
- Aid healthcare professionals in the thorough and efficient evaluation and management of CPP.
- Facilitate education of healthcare professionals, trainees, and patients.
- Create a framework to aid research design, implementation, and analysis.
- Provide policymakers with more precise terminology for resource allocation.^8^

The primary objective of this project was to respond to the FIGO‐IPPS call to action^8^ and develop a pragmatic classification system for conditions associated with CPP that attempts to fulfill these requirements. We aimed to create a classification system for CPP that mirrors existing successful models for classifying conditions with similar impacts. A predicate example can be found in the widely recognized FIGO system for characterizing the symptoms and classifying the causes of non‐gestational abnormal uterine bleeding (AUB) in the reproductive years. This classification system, known by the acronym PALM‐COEIN, has successfully transformed education, research, and the diagnosis and management of AUB by standardizing terminology and guiding healthcare practices.^11^, ^12^

Although we acknowledge the potential challenges to creating a practical but comprehensive classification system for CPP, we recognize several parallels between CPP and AUB, as both conditions are associated with multiple possible causes and comorbidities, impact a large proportion of the global female population, and profoundly and negatively affect women's lives and well‐being.^5^, ^8^, ^12^, ^13^ Furthermore, developing a comprehensive classification system for CPP, analogous to that for AUB, requires collaboration from diverse stakeholders, including international medical specialty and subspecialty societies, patients, individuals with personal experience of pain, advocacy groups, researchers, healthcare professionals, and educators.^8^ Therefore, by engaging these diverse stakeholders, this project aims to establish a practical classification system that standardizes CPP terminology, enhances education, and improves global diagnosis and management.

## 2 METHODS

To establish consensus on the CPP classification system, we used a version of the RAND/UCLA Delphi consensus development methodology that has been used to develop other FIGO‐supported projects in women's reproductive health.^14^, ^15^, ^16^, ^17^ The Delphi process comprises a structured, systematic, yet iterative, and largely anonymous consensus approach involving multiple rounds of online electronic surveys and, where appropriate, facilitated discussion among a group of stakeholders conducted over a period of time.^16^ Initially established in the 1950s by the RAND Corporation to develop military strategy,^14^, ^18^ multiple iterations have been used across a spectrum of disciplines, and the process has evolved into a generally accepted method in medicine for developing consensus statements and clinical guidelines.^15^ The ability to participate anonymously and to involve a spectrum of stakeholders is thought to strengthen the value of the output, thereby improving the design and interpretation of research, the effectiveness of teaching, and the quality of clinical care.^14^, ^15^

### 2.1 Steering committee formation and responsibilities

A steering committee (SC) was formed, comprising eight experts in CPP and members of key international medical societies. There were two members from each of the two organizations developing this system; MGM and JDVE from FIGO, and GL and JC from the International Pelvic Pain Society (IPPS). The members of the SC also held membership in the following organizations: four (AS, GL, JDVE, MGM) from the AAGL, three (GL, MGM, SK) from the American College of Obstetricians and Gynecologists (ACOG), one (CA) from the Society of Obstetricians and Gynaecologists of Canada (SOGC), one (SA) from the American Society of Reproductive Medicine (ASRM), one (AWH) from the European Society of Human Reproduction and Embryology (ESHRE), and one (AWH) from the World Endometriosis Society (WES). Notably, several of the SC were members of more than one relevant society or organization.

The SC obtained approval to proceed from the FIGO Education Communication and Advocacy Committee (ECAC) and the IPPS Executive Board. The SC's function was to manage the Delphi process, including the identification of stakeholders, the design and testing of surveys, the collection and analysis of data, maintaining appropriate communication with the participants, designing the systems according to input from panelists, obtaining consensus support for the developed systems, and, finally, the creation and submission of a manuscript for publication. Initial SC responsibilities included defining the scope and objectives of the project. Two primary objectives were established: first, to determine if there is a need for a classification system to facilitate clinical care, trainee education, and CPP research (including its design, conduct, and interpretation); and second, if such a need exists, to develop or adopt standardized systems for classifying conditions associated with CPP.

### 2.2 Stakeholder selection and engagement

FIGO is an international organization comprising the national gynecological and obstetrical societies from approximately 139 countries, and each was contacted and invited to participate in the Delphi process. The SC further identified relevant international stakeholder societies regardless of their core discipline and solicited their participation via an appropriate representative. Beyond the national, subspecialty, and other societies, the SC identified relevant journals and internationally recognized experts primarily based on their contributions to the literature. The SC also identified lay societies and patients to allow the inclusion of the lay stakeholder population. All who responded and expressed interest were sent formal invitations with a more detailed project description. Consequently, invitations were sent to 114 stakeholders, including clinicians (e.g. physicians and nurses) from various specialties (e.g. gynecology, urology, gastroenterology, and primary care), relevant medical societies, lay people, individuals living with the experience of pain, researchers, pelvic pain experts, educators, and journal editors. Of those invited, 77 (67.5%) confirmed their participation and were invited to attend a virtual orientation meeting to discuss project objectives, the Delphi process, and consensus criteria.

### 2.3 Delphi process and consensus criteria

The modified Delphi system was similar to the original developed by the RAND Corporation^14^, ^18^ and the one used in previous FIGO consensus‐development processes.^11^ The SC developed a series of statements to elicit the panel's opinions regarding CPP issues. Each statement was accompanied by a 9‐point Likert scale (1 = strongest disagreement, 9 = strongest agreement). Each survey round comprised several statements, and each round was tested internally by the SC until deemed satisfactory to distribute to the panel members. All surveys were created using SurveyMonkey® (San Mateo, CA, USA) and distributed electronically using the email addresses supplied by the participants. Three email reminders were sent to non‐respondents.

The responses were collected electronically and anonymously using the SurveyMonkey platform, with data aggregated and converted into MS Excel (Microsoft Corp., Redmond, WA, USA). For each statement in the survey, a mean score ≥7 with <15% disagreement (score ≤3) defined consensus agreement, while a mean score ≤3 with <15% agreement (score ≥7) would be regarded as consensus disagreement. All other results were considered indeterminate. Participants were informed that indeterminate statements would be refined based on their feedback and re‐presented in subsequent rounds. This iterative process would continue until consensus was achieved or unattainable by round 3.

### 2.4 Iterative survey rounds and data analysis

The SC developed the initial round 1 survey consisting of 45 statements (Table 1), disseminated the anonymous electronic survey, collected responses, and analyzed the data. For statements without consensus, participant responses were used to refine and create round 2, which comprised 13 statements. Responses from the second round were used to develop an initial draft of the classification system, which was then distributed to all participants for comment and approval in round 3. In this round, additional statements were discussed and clarified before creating the next version of the classification system. Round 3 was conducted via virtual meeting using Zoom (Zoom Communications Inc., San Jose, CA, USA) to facilitate discussion and clarification of each statement with the stakeholders. When necessary, respondents were provided with research and evidence‐based guidelines to help them make informed decisions. The SC used this feedback to make final decisions on the structure and content of the classification system and reviewed the final system for clarity, accuracy, and applicability. The SC also prepared all presentation materials and manuscripts for publication with input from the participating stakeholders.

**TABLE 1:** Statements disseminated in Delphi rounds 1–3.

Round 1 statements	
1	What is your geographic location?
2	What is your age?
3	What is your gender?
4	Who/What do you represent in this process? If you are not a designated journal, subspecialty, or national society representative, you are likely an “Expert‐at‐Large.”
5	What is your main area of expertise?
6	Optional. Select a second area of expertise
7	Chronic pelvic pain is a common problem in the country where I live.
8	Chronic pelvic pain may contribute to poor physical health.
9	Chronic pelvic pain may contribute to poor quality of life.
10	Chronic pelvic pain may negatively affect the social or personal relationships of those who experience it.
11	Chronic pelvic pain may have a negative economic impact on those who experience this type of pain.
12	Chronic pelvic pain may negatively affect cognition such as thinking, perceiving, and reasoning.
13	Chronic pelvic pain may negatively affect sexual health.
14	There are multiple gynecologic conditions that may contribute to chronic pelvic pain
15	There are multiple NON‐gynecologic conditions that may contribute to chronic pelvic pain.
16	Sometimes there is no obvious contributor or trigger for chronic pelvic pain.
17	Chronic pelvic pain may be provoked by specific physical activities or events: sexual intercourse, defecation, urination.
18	Endometriosis may contribute to chronic pelvic pain.
19	Adenomyosis may contribute to chronic pelvic pain.
20	Other pelvic conditions, such as fibroids and ovarian cysts, may contribute to chronic pelvic pain.
21	Gastrointestinal disorders may contribute to chronic pelvic pain.
22	Disorders of the urinary tract may contribute to chronic pelvic pain.
23	Vaginal disorders may contribute to chronic pelvic pain.
24	Vulvar disorders may contribute to chronic pelvic pain.
25	Vascular disorders may contribute to chronic pelvic pain.
26	Neurologic disorders may contribute to chronic pelvic pain.
27	Myofascial disorders may contribute to chronic pelvic pain.
28	There is no universally agreed‐upon definition of chronic pelvic pain; it is defined in different ways by various societies and guidelines.
29	I agree with the ACOG (American College of Obstetricians & Gynecologists) definition: “Chronic pelvic pain consists of pain symptoms perceived to originate from pelvic organs or structures typically lasting more than 6 months. It is often associated with negative cognitive, behavioral, sexual, and emotional consequences and with symptoms suggestive of lower urinary tract, sexual, bowel, pelvic floor, myofascial, or gynecological dysfunction.”
30	I agree with the IASP (International Association for the Study of Pain) definition of chronic pelvic pain: “Chronic pelvic pain is defined as chronic or persistent pain perceived in structures related to the pelvis of either men or women. It is often associated with negative cognitive, behavioral, sexual and emotional consequences as well as with symptoms suggestive of lower urinary tract, sexual, bowel, pelvic floor or gynecological dysfunction.”
31	I agree with the RCOG (Royal College of Obstetricians and Gynaecologists) definition of chronic pelvic pain: “Chronic pelvic pain can be defined as intermittent or constant pain in the lower abdomen or pelvis of a woman of at least 6 months duration, not occurring exclusively with menstruation or intercourse and is not associated with pregnancy.”
32	A well‐designed, internationally accepted classification of chronic pelvic pain would assist clinicians in evaluating patients.
33	A well‐designed, internationally accepted classification of chronic pelvic pain would assist clinicians to select effective treatments for patients with chronic pelvic pain.
34	A well‐designed, internationally accepted classification of chronic pelvic pain, would help educators teach about pelvic pain.
35	A well‐designed, internationally accepted classification of chronic pelvic pain would help researchers develop and interpret studies, such as meta‐analysis and clinical trials.
36	A well‐designed, internationally accepted classification of chronic pelvic pain would help researchers design and interpret epidemiological research such as prevalence and population studies.
37	Chronic pelvic pain should be renamed persistent pelvic pain.
38	Chronic pelvic pain may be intermittent.
39	Chronic pelvic pain may be cyclical – associated with specific times in the menstrual cycle.
40	Chronic pelvic pain may be located in the lower abdomen or pelvis.
41	Chronic pelvic pain may be located in the genitalia, including the vulva.
42	Chronic pelvic pain may be located in the perineum, including the anal and lower buttocks area.
43	Chronic pelvic pain can include pain that is located in the posterior back area.
44	Chronic pelvic pain is a pain that lasts longer than six (6) months.
45	Chronic pelvic pain is a pain that lasts longer than three (3) months.

## 3 RESULTS

The SC was finalized in December 2022, and its first formal meeting was convened on December 14, 2022. Subsequent meetings have been held twice monthly, and the SC's work remains ongoing.

### 3.1 Stakeholder composition and magnitude of the problem

The round 1 survey was distributed to all 77 stakeholders who agreed to participate. A total of 65 (84.4%) stakeholders responded in round 1, of whom 64 provided complete responses and one returned an incomplete survey. The stakeholders represented are detailed in Table 2. All responses, including incomplete ones, were included in the analysis. All round 1 respondents were invited to participate in round 2, which involved a survey containing 13 additional statements. A total of 54 (70.1%) stakeholders completed round 2, and these responses were analyzed to create three additional statements for round 3. All round 2 participants were invited to round 3, and 34 (44.2%) stakeholders participated in this round. During the meeting, survey statements were discussed in detail, followed by electronic and anonymous responses from the participants.

**TABLE 2:** Stakeholders involved in the Delphi process.

Name	Stakeholder representation
Chronic Pain Research Alliance	Patient advocacy
Endometriosis UK	Patient advocacy
International Painful Bladder Foundation	Patient advocacy
National Vulvodynia Association	Patient advocacy
Pelvic Pain Support Network	Patient advocacy
Archives of Gynecology and Obstetrics	Medical journal
British Journal of Obstetrics & Gynaecology	Medical journal
Journal of Minimally Invasive Gynecology	Medical journal
Obstetrics and Gynecology	Medical journal
Reproduction	Medical journal
Reproduction and Fertility	Medical journal
American College of Obstetricians and Gynecologists	International society representing healthcare professionals
Colombian Federation of Obstetrics and Gynecology	International society representing healthcare professionals
Congolese Society of Obstetricians‐Gynecologists	International society representing healthcare professionals
Ethiopian Society of Obstetricians and Gynecologists	International society representing healthcare professionals
Iraqi Society of Obstetrics & Gynecology	International society representing healthcare professionals
Kenya Obstetrical and Gynaecological Society	International society representing healthcare professionals
Kyrgyz Association of Obstetricians, Gynecologists and Neonatologists	International society representing healthcare professionals
Österreichische Gesellschaft für Gynäkologie und Geburtshilfe (Austrian Society of Gynaecology and Obstetrics)	International society representing healthcare professionals
Royal College of Obstetricians and Gynaecologists	International society representing healthcare professionals
Sierra Leone Association of Gynecologists and Obstetricians	International society representing healthcare professionals
Sociedad Chilena de Obstetricia y Ginecología	International society representing healthcare professionals
Society of Obstetricians & Gynaecologists of Pakistan	International society representing healthcare professionals
Spanish Society of Obstetrics and Gynecology	International society representing healthcare professionals
The Society of Obstetricians and Gynaecologists of Canada	International society representing healthcare professionals
AAGL^a^	International medical subspecialty society representing healthcare professionals
Academy of Pelvic Health Physical Therapy	International medical subspecialty society representing healthcare professionals
American Urogynecologic Society	International medical subspecialty society representing healthcare professionals
Brazilian Society of Laparoscopic and Robotic Surgery	International medical subspecialty society representing healthcare professionals
Canadian Society for the Advancement of Gynecologic Excellence	International medical subspecialty society representing healthcare professionals
Colorado Center for Behavioral Medicine	International medical subspecialty society representing healthcare professionals
Convergences In Pelviperineal Pain	International medical subspecialty society representing healthcare professionals
European Endometriosis League	International medical subspecialty society representing healthcare professionals
European Society of Human Reproduction and Embryology	International medical subspecialty society representing healthcare professionals
International Association for the Study of Pain ‐ Abdominal and Pelvic SIG	International medical subspecialty society representing healthcare professionals
International Pelvic Pain Society	International medical subspecialty society representing healthcare professionals
International Society of Neuropelviology	International medical subspecialty society representing healthcare professionals
International Society of Physical and Rehabilitation Medicine	International medical subspecialty society representing healthcare professionals
International Urogynecological Association × 2	International medical subspecialty society representing healthcare professionals
Multidisciplinary Approach to the Study of Chronic Pelvic Pain Research Network	International medical subspecialty society representing healthcare professionals
Office of Women's Health, Central VA Office	International medical subspecialty society representing healthcare professionals
Pudendal Neuralgia Association	International medical subspecialty society representing healthcare professionals
Sociedad Espanola para el Estudio de los Miomas y la Endometriosis	International medical subspecialty society representing healthcare professionals
Society of Endometriosis and Uterine Disorders	International medical subspecialty society representing healthcare professionals
Society of Laparoscopic and Robotic Surgeons	International medical subspecialty society representing healthcare professionals
United States Association for the Study of Pain	International medical subspecialty society representing healthcare professionals
University of Rome Tor Vergata, Department of Surgical Sciences, Ob/Gyn Unit	International medical subspecialty society representing healthcare professionals

^a^ Formerly the American Association of Gynecologic Laparoscopists.

**FIGURE 1:**
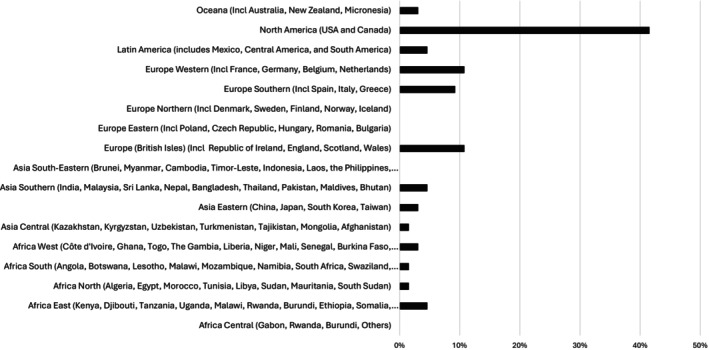
Geographic distribution of stakeholder location (*n* = 65). Note: Where the row is incomplete (…) the full contents are as follows: Africa West (Côte d’Ivoire, Ghana, Togo, The Gambia, Liberia, Niger, Mali, Senegal, Burkina Faso, Sierra Leone, Guinea, Guinea‐Bissau, Benin, Nigeria, Cabo Verde, Chad, Cameroon, Sāo Tomé and Principe, Equatorial Guinea, Central African Republic, Gabon, Namibia, Democratic Republic of the Congo, Angola, Libya, Morocco, Republic of the Congo) Asia South‐Eastern (Brunei, Myanmar, Cambodia, Timor‐Leste, Indonesia, Laos, the Philippines, Singapore, Thailand, Vietnam) Africa South (Angola, Botswana, Lesotho, Malawi, Mozambique, Namibia, South Africa, Swaziland, Zambia, Zimbabwe) Africa East (Kenya, Djibouti, Tanzania, Uganda, Malawi, Rwanda, Burundi, Ethiopia, Somalia, Comoros, Mozambique, Seychelles, Madagascar, Mauritius, Réunion, Eritrea, Mayotte) [Correction added on 24 October 2025, after first online publication: Figure 1 was incorrect and has been replaced in this version.]

**FIGURE 2:**
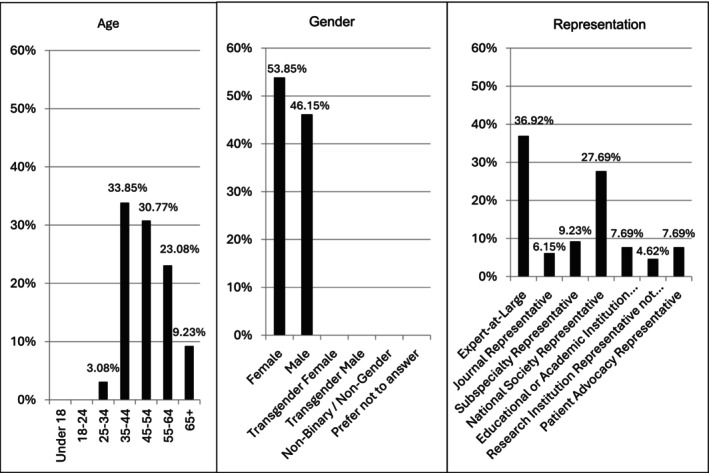
Demographic characteristics and representation of stakeholders (*n* = 65).

The demographic and group representation of the respondents is shown in Figures 1 and 2. Of the respondents, 43 (66.2%) reported involvement in direct clinical care, 13 (20.0%) in clinical research, 2 (3.1%) in advocacy, and 2 (3.1%) reported being an individual living with CPP. Notably, many stakeholders had multiple areas of expertise, including clinicians who were also involved in research (40.3%) and teaching (14.5%). Most stakeholders agreed that a well‐designed, internationally accepted classification of CPP would be beneficial for clinicians in evaluating patients (96.9% agreement, means score = 8.3) and selecting effective treatments for patients with CPP (92.3% agreement, mean score = 7.9), for educators teaching about CPP (93.8% agreement, mean score = 8.3), and for researchers when developing and interpreting studies including meta‐analysis (93.8% agreement, means score = 8.2) and epidemiologic research including prevalence and population studies (96.9% agreement, mean score = 8.3).

### 3.2 Establishing consensus on a definition for CPP

For each statement in the survey, we analyzed the level of agreement using the mean Likert scale score (1 = strongest disagreement, 9 = strongest agreement) and the percentage of respondents in agreement (Likert score ≥7) or disagreement (Likert score ≤3). The participants strongly agreed that CPP is a common problem in their country (93.8% agreement, mean score = 7.9). There was also consensus that CPP is a significant problem contributing to poor physical health (96.9% agreement, mean score = 8.3), quality of life (98.5% agreement, mean score = 8.6), and interference with social or personal relationships (98.5% agreement, mean score = 8.6). Participants also agreed that CPP may have a negative economic impact (96.9% agreement, mean score = 8.2). In addition, there was widespread agreement that CPP may negatively affect cognition (90.8% agreement, mean score = 7.8) and sexual health (98.5% agreement, mean score = 8.6), and that multiple gynecologic (98.5% agreement, mean score = 8.3) and non‐gynecologic (96.9% agreement, mean score = 8.1) conditions may contribute to CPP. Although 73.8% agreed (mean score = 7.2) that sometimes there is no obvious contributor or trigger for CPP, there was strong consensus that CPP may be provoked by specific physical activities such as sexual intercourse, defecation, and urination (96.9% agreement, mean score = 8.4).

The group also agreed that CPP might be classified as intermittent (93.8% agreement, mean score = 7.8), cyclical if repetitively associated with specific times in the menstrual cycle (98.1% agreement, mean score = 7.8), and located in various areas of the lower abdomen or pelvis (96.9% agreement, mean score = 8.3). Further exploration of pain locations revealed agreement that pelvic pain may also be located in the genitalia, including the vulva (79.7% agreement, mean score = 7.4) and perineum, including the lower buttocks and anal region (73.4% agreement, mean score = 7.2). However, in round 1, there was notable disagreement regarding lower back pain within the definition of CPP (20.3% disagreement, mean score = 6.3), especially if the back pain was not accompanied by anterior pelvic pain. Consequently, this question was further refined in rounds 2 and 3 after a video detailing the standardized definition of the pelvis was provided to the stakeholders. Subsequently, the participants determined that low back pain, in the absence of anterior pelvic pain, should be excluded from the definition of CPP. Furthermore, they agreed that the CPP definition and classification system should adhere to established pelvic terminology,^19^ defining the pelvis as the anatomical region between the lumbar area of the lower abdomen and the thighs,^19^ containing the pelvic cavity and the perineum. This region encompasses the bony pelvis (bordered by the pubis, ischium, ilium, coccyx, and sacrum), the pelvic cavity (containing musculoskeletal, vascular, and neurological structures, and viscera such as the uterus, ovaries, fallopian tubes, vagina, bladder, pelvic colon, rectum, and anus), and the perineum (including the urethra, vaginal introitus, anal canal, and perineal body).^19^

For earlier definitions of CPP, 78.5% (mean score = 7.0) of participants agreed that there is no universally agreed‐upon definition of CPP. When presented with definitions of CPP established by ACOG, the International Association for the Study of Pain (IASP), and the Royal College of Obstetricians and Gynaecologists (RCOG), stakeholders generally agreed with definitions proposed by ACOG (80.0% agreement, mean score = 6.9) and IASP (83.1% agreement, mean score = 7.2). However, there was a high percentage of disagreement (20.0% disagreement, mean score = 5.9) with the RCOG definition.^19^ The primary focus of disagreement centered around the duration of pain, with the ACOG and IASP definitions allowing for a more flexible timeframe. Specifically, ACOG describes CPP as pain “typically” lasting longer than 6 months.^3^ In contrast, IASP does not specify a duration, while RCOG requires a pain duration of at least 6 months.^20^ In the initial round, the stakeholders could not agree on whether the duration of CPP should be strictly limited to longer than 3 or 6 months. However, they considered temporality important when evaluating the etiology of pain and distinguishing between acute and chronic pain. Conversely, they also considered the potential for overlooking features of chronic pain (e.g. negative impact on quality of life, sensitization) even in cases with a duration of less than 6 months. Ultimately, the group agreed that the duration of pain should be flexible, with language describing the CPP as having a typical duration of 3 months or more (82.4% agreement, mean score = 7.4).

Participants were asked whether CPP should be renamed “persistent pelvic pain.” Here, there was significant disagreement (35.9% disagreement, mean score = 5.2) among stakeholders. Although such a change may reduce the stigma associated with CPP, participants expressed concern that altering terminology could lead to confusion in clinical practice and research.

The final consensus was that CPP should be defined as pain localized to the pelvis that may be cyclical or non‐cyclical and typically lasts 3 months or longer after failed initial therapies. The pain can significantly impact societies and individuals; however, on an individual level, the pain may be associated with disability, distress, and negative cognitive, behavioral, sexual, emotional, and economic consequences. The pain may present with symptoms of musculoskeletal, urinary, bowel, reproductive, vascular, and neurologic dysfunction, such as urgency, frequency, abnormal bowel movements, dysmenorrhea, allodynia, hyperalgesia, vascular insufficiency, and AUB. The inclusion of “after failed initial therapies” emphasizes that pelvic pain should not be deemed chronic without first attempting suitable initial treatments. Furthermore, conditions that can cause both acute and chronic pelvic pain, like pelvic inflammatory disease (PID), should not be categorized within this classification system unless initial treatments prove unsuccessful.

### 3.3 Selecting conditions to be included in the classification system

In rounds 1 and 2, the group agreed that several conditions should be included in the classification system because they may contribute to CPP. These included gastrointestinal disorders (95.4% agreement, mean score = 7.9), conditions of the urinary tract (93.8% agreement, mean score = 8.1), vaginal disorders (87.7% agreement, mean score = 7.6), vulvar conditions (89.2% agreement, mean score = 7.6), vascular disorders (72.3% agreement, mean score = 7.2), neurologic disorders (95.4% agreement, mean score = 8.0), musculoskeletal disorders (93.8% agreement, mean score = 8.1), and gynecologic disorders such as endometriosis (96.9% agreement, mean score = 8.4), adenomyosis (89.3% agreement, mean score = 8.0), and dysmenorrhea (agreement 82.5%, mean score = 7.5). There was a high level of neutrality as to whether leiomyoma (49.1% agreement, 26.3% neutral, mean score = 5.8), ovarian cysts not associated with endometriosis (38.6% agreement, 19.3% neutral, mean score = 4.8), chronic pelvic inflammatory disease (73.7% agreement, 12.3% neutral, mean score = 6.9), and vaginal pain with touch or penetration should be included (56.1% agreement, 22.8% neutral, mean score = 6.3). However, to stay consistent with published research and consensus statements^21^ demonstrating that these conditions can be associated with CPP, the SC decided to include them in the “R – reproductive” category of the classification system.^3^, ^21^

There was agreement on including a “no pain contributor identified” category for use when pain cannot be attributed to a known pain contributor despite a comprehensive, meticulous, and exhaustive evaluation and diagnostic testing (77.2% agreement, mean score = 7.1). Similarly, the group considered it essential to include a category for “not otherwise classified” for cases where an underlying condition associated with the pain can be identified but is not explicitly listed in the classification system (70.2% agreement, mean score = 6.8). It must be emphasized that the evaluator's lack of familiarity with the multiple conditions and comorbidities associated with CPP does not justify assigning a “no pain contributor identified” or “not otherwise classified” label. In such cases, a referral to a pelvic pain specialist is more appropriate. Lastly, there was a consensus to include central sensitization/nociplastic as an independent category in the classification system (71.9% agreement, mean score = 7.0).

### 3.4 Development of the classification system

**FIGURE 3:**
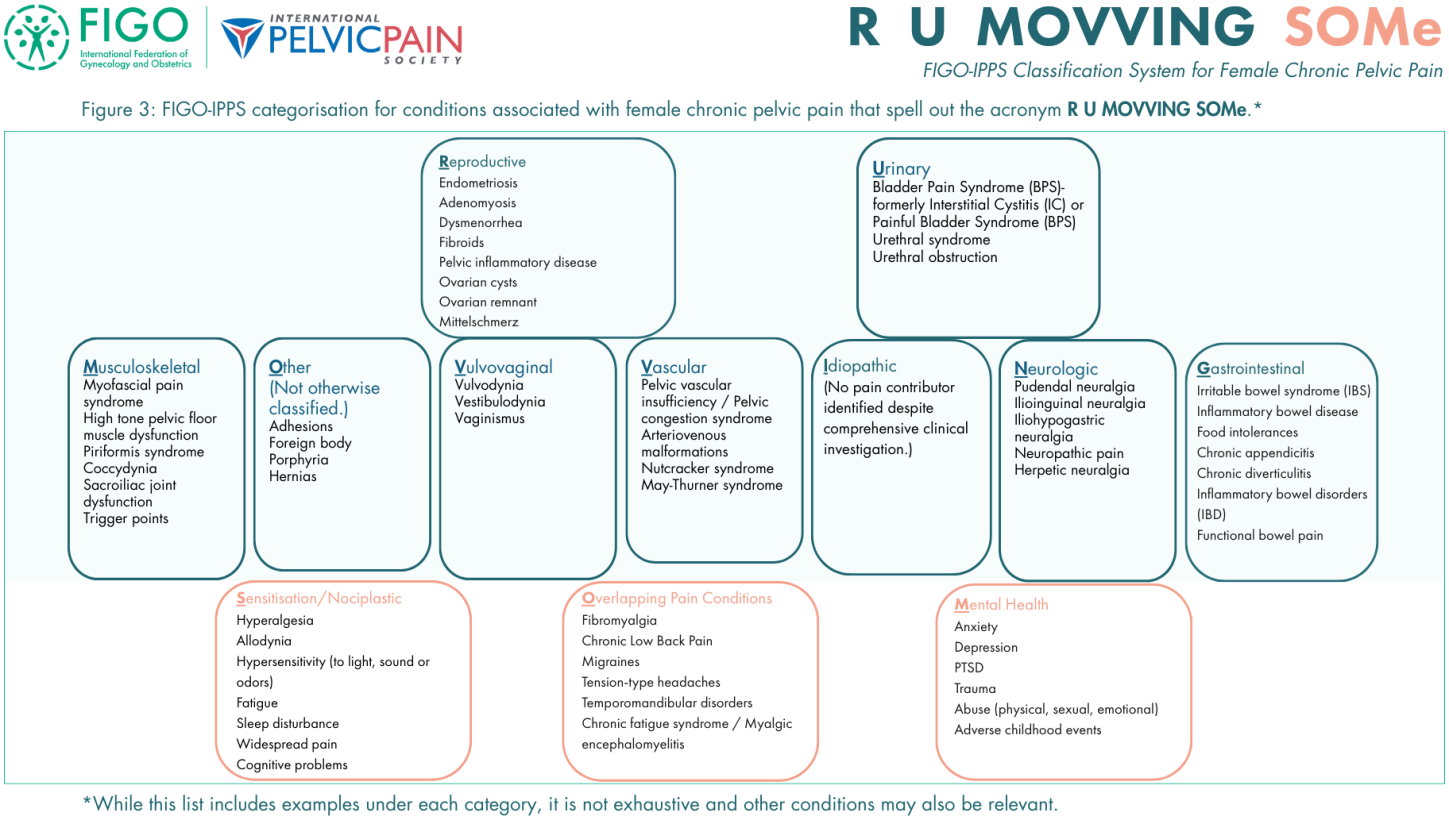
FIGO‐IPPS categorization for conditions associated with female chronic pelvic pain that spell out the acronym R U MOVVING SOMe.* *While this list includes examples under each category, it is not exhaustive and other conditions may also be relevant. [Correction added on 16 September 2025, after first online publication: Figure 3 was incorrect and has been replaced in this version.]

In total, 12 system categories were identified and arranged to spell out the acronym R U MOVVING SOMe (pronounced “Are you moving some”). The acronym letters represent conditions from the following categories: Reproductive, Urinary, Musculoskeletal, Other (not otherwise classified), Vulvovaginal, Vascular, Idiopathic (no pain contributor identified), Neurologic, Gastrointestinal, Sensitization/Nociplastic, Overlapping pain conditions, and Mental health (Figure 3).

The R U MOVVING categories typically represent conditions originating from specific peripheral organ systems. In contrast, SOMe conditions are rarely categorized as primary pain generators; instead, they predominantly function as modifiers or intensifiers of pain in the presence of co‐existing organ system pathology. For example, anxiety may serve as a pain exacerbator while co‐occurring with endometriosis, which would be classified as the primary pain etiology. In such a scenario, the relevant “R” (e.g. Reproductive/Gynecological) and “Me” (e.g. Mental health) categories would necessitate comprehensive documentation and assessment.

### 3.5 R – Reproductive

This category encompasses conditions that primarily originate from the uterus, ovaries, fallopian tubes, and vagina. Earlier studies indicate that approximately 50% of reproductive‐aged women with CPP report a gynecologic origin for their pain.^22^ More recent population‐based research has identified the most common gynecologic diagnoses reported by women with CPP as endometriosis (23.4%)^23^ and ovarian cysts (23.0%), with less frequent diagnoses including pelvic inflammatory disease (3.3%) and vulvodynia (1.6%).^3^, ^5^, ^6^, ^21^, ^23^ This category also includes conditions that may not consistently present with CPP, such as adenomyosis^24^, ^25^, ^26^ and leiomyoma,^27^, ^28^ and less common conditions, including ovarian remnant, and Mittelschmerz syndrome.^3^, ^21^ Conditions in this category significantly contribute to CPP through various pathophysiological mechanisms, including inflammation, tissue distortion, hormonal influences on tissue growth and sensitivity, and potential organ dysfunction. Dysmenorrhea, or cyclical pelvic pain associated with menstruation, is a common feature of many of these conditions, although it may also be classified as a separate CPP‐associated condition.^3^, ^21^, ^29^

### 3.6 U – Urinary

This category includes conditions related to the urinary tract, including the bladder, ureters, and kidneys. The most prevalent example is interstitial cystitis/bladder pain syndrome (IC/BPS), a condition defined by the American Urological Association as pelvic pain, pressure, or discomfort related to urinary bladder function (filling and/or emptying) lasting longer than 6 weeks, in the absence of infection or other identifiable causes.^30^, ^31^, ^32^ IC/BPS is estimated to affect 3%–6% of all women in the USA, although prevalence estimates are imprecise and its incidence remains unknown.^31^, ^33^, ^34^ Other conditions within this category include urethral syndrome, characterized by chronic inflammation of the urethra, and bladder outlet obstruction, such as urethral stricture. These conditions can cause pelvic pain through several mechanisms, including inflammation of the urothelium, irritation of the urinary tract lining, smooth muscle spasm, and altered bladder function, leading to urgency, frequency, distention, and discomfort during voiding and/or bladder filling. IC/BPS, in particular, is a common overlapping condition in which sensitization/nociplastic pain is the known mechanism for pain.^35^, ^36^, ^37^ Typical urinary tract infections and kidney, bladder, or urethral stones are generally acute conditions. They should not be categorized as CPP conditions unless they do not respond to standard treatment and are associated with persistent pain and/or significant signs of impaired quality of life, poor mental health, and disability.^33^

### 3.7 M – Musculoskeletal

This category can describe disorders of the muscles, bones, and connective tissues of the pelvis. It may include dysfunction, instability, or abnormal movement of the coccyx, pubic symphysis, and hip and sacroiliac joints.^38^, ^39^ The anterior abdominal wall muscles, including the right and left inguinal regions, and the deep pelvic muscles (e.g. piriformis, levators) may also be affected.^38^, ^40^ This category may also include high tone pelvic floor dysfunction characterized by hypertonicity or impaired relaxation.^39^ Altered biomechanics, focal areas of tenderness (e.g. trigger points) leading to nerve compression and/or sensitization, and inflammation of supporting structures are some of the features of conditions included in this category.^41^

### 3.8 O – Other (not otherwise classified)

This category may be used for conditions that currently do not fit clearly into other categories of the classification system but are suspected of being associated with the patient's pain, such as adhesions, retained foreign bodies, and porphyria.^5^, ^21^ This category may also include conditions such as hernias if associated with chronic pain localized to the abdominal wall.^28^, ^42^, ^43^, ^44^ Selection of this category does not diminish the significance of the patient's pain; instead, it acknowledges the complexity or lack of precise classification at this time. Importantly, conditions in this category may be reclassified as new information becomes available.^12^

### 3.9 V – Vulvovaginal

This category is limited to conditions that result in persistent pain localized to the external genitalia, specifically the vaginal introitus, vulva, clitoris, and vestibular bulbs. The primary condition within this category is vulvodynia, which, as defined by the International Society for the Study of Vulvovaginal Disease (ISSVD) in collaboration with the International Society for the Study of Women's Sexual Health (ISSWSH) and the International Pelvic Pain Society (IPPS), is vulvar pain with a duration of at least 3 months without a clear identifiable cause that may have potential associated factors.^45^, ^46^, ^47^ Vulvodynia can be further characterized by its anatomical distribution (localized to specific vulvar structures, such as the labia minora, labia majora, vestibule, and clitoris, or generalized, affecting the entire vulva) and its temporal pattern and triggers (provoked, spontaneous, primary onset with first penetration, secondary onset after a pain‐free period, intermittent, or constant).^45^, ^46^, ^47^, ^48^

It is important to note that the ISSVD/ISSWSH/IPPS classification system also addresses vulvar pain associated with identifiable underlying medical conditions.^45^, ^46^, ^47^, ^48^ This includes infection (e.g. herpes, candidiasis), inflammatory dermatoses (e.g. lichen sclerosus, lichen planus), neoplastic disorders (e.g. Paget disease, squamous cell carcinoma), neurologic conditions (e.g. post‐herpetic neuralgia), trauma from obstetrical events or genital mutilation, iatrogenic causes (postoperative pain, chemotherapy, radiation), and hormonal deficiencies (genitourinary syndrome of menopause, vulvovaginal atrophy, lactational amenorrhea).^45^, ^46^, ^47^, ^48^ In such instances, clinicians can use the ISSVD/ISSWSH/IPPS categories as a second subclassification system.^47^ For example, chronic vulvar pain resulting from lichen sclerosus would be classified under a vulvovaginal disorder using R U MOVVING SOMe and further subclassified as “inflammatory” within the ISSVD/SSWSH/IPPS framework.^47^, ^49^

### 3.10 V – Vascular

This category encompasses conditions affecting the blood vessels in the pelvic region. A primary example is pelvic vascular insufficiency (PVI), commonly known as pelvic congestion syndrome (PCS), characterized by dilated and tortuous veins in the pelvis that can lead to chronic pain.^43^, ^50^ Although the definition and pathophysiology of PCS/PVI continue to be investigated, increasing evidence supports its association with CPP, and therapeutic non‐evidence‐based interventions are available.^43^, ^51^ This category includes arteriovenous malformations (AVMs) and other vascular conditions such as vascular compression syndromes (e.g. Nutcracker syndrome, May‐Thurner syndrome) associated with CPP.^50^ Pain in these conditions can arise from altered blood flow dynamics, venous engorgement, hypoxia, and increased pressure on surrounding pelvic structures.^50^, ^52^ Including a vascular category in the R U MOVVING SOMe classification system was based on consensus agreement between the stakeholders and the SC.

### 3.11 I – Idiopathic (no pain contributor identified)

This classification is reserved for instances of persistent pelvic pain where a definitive underlying pathological process or specific etiological factor cannot be identified despite a comprehensive and systematic clinical investigation.^5^, ^21^ This necessitates the utilization of thorough history taking, meticulous physical examination, appropriate imaging techniques (e.g. ultrasonography, magnetic resonance imaging [MRI], computed tomography), and other relevant diagnostic modalities (e.g. laboratory analyses, endoscopic procedures) as clinically indicated.^3^, ^21^ The assignment of the “Idiopathic” category signifies that a condition or reason for the patient's pelvic pain remains elusive based on current medical knowledge and available diagnostic capabilities. Although an idiopathic diagnosis can be a source of frustration for both the individual experiencing pain and the clinicians involved in their care, it is a necessary category that acknowledges the inherent limitations of current medical understanding and diagnostic technologies.^5^

We emphasize that the “Idiopathic” category must not be used in cases where the clinical evaluation is incomplete. Regardless of the accessibility or availability of evaluation resources, this category should only be considered after a rigorous and comprehensive diagnostic workup has been undertaken to exclude identifiable organic pathologies. Prematurely assigning the “Idiopathic” label without a thorough investigation could lead to misclassifying conditions with potentially treatable underlying causes and may hinder the development of appropriate management strategies. Therefore, this category serves as a diagnosis of exclusion, made only after comprehensive efforts to identify a specific etiology have been exhausted.^53^, ^54^

### 3.12 N – Neurologic

This category focuses on conditions that affect the peripheral and central nervous system (CNS) structures innervating the pelvis. This category can include conditions affecting the iliohypogastric, ilioinguinal, genitofemoral, obturator, pudendal, and posterior femorocutaneous nerves and their branches.^3^, ^5^, ^21^, ^42^, ^55^ Pudendal neuralgia, characterized by neuropathic pain, numbness, or paresthesia in the distribution of the pudendal nerve branches, is often attributed to nerve compression, entrapment, or irritation along its course.^56^, ^57^, ^58^ Neuropathy refers to a broader category involving damage (e.g. postoperative neuropathy) or dysfunction of peripheral nerves within the pelvis, which can arise from various etiologies, including metabolic disorders, infections, trauma, or idiopathic causes. Referred pain originating from other neural structures, such as the lumbar spine (e.g. lumbar radiculopathy and plexopathy), can also manifest as pelvic pain due to the convergence of sensory pathways within the CNS.^59^ Diagnoses within this neurologic category are often initially based on characteristic clinical presentations, including the anatomical distribution of pain (e.g. following specific cutaneous innervation or nerve pathways), the quality of the pain (e.g. burning, lancinating, tingling), and associated neurological symptoms (e.g. weakness, sensory loss).^5^, ^21^, ^59^ The physical examination findings are often characteristic, with allodynia and hyperalgesia being consistent findings. Nerve blocks are frequently utilized for diagnostic and therapeutic purposes.^56^, ^60^ However, this classification inherently assumes that clinically indicated diagnostic testing has been conducted to identify other disorders that may mimic neuropathic pain. Such investigations may include, but are not limited to, nerve conduction studies (NCS) and electromyography (EMG) to assess peripheral nerve function, as well as imaging modalities like MRI of the spine and pelvis to rule out structural lesions (e.g. disc herniations, tumors) that could be compressing or irritating nerve roots or peripheral nerves.^42^, ^60^ These specific etiologies, if identified and deemed the primary cause of pain, would typically be addressed acutely and, if responsive to therapy, would not be categorized within this classification system.

### 3.13 G – Gastrointestinal

This category describes conditions related to the digestive system that can manifest as CPP, often associated with bloating and bowel dysfunction. The most common condition included in this category is irritable bowel syndrome (IBS).^5^, ^21^, ^61^ This chronic functional gastrointestinal disorder often presents with pain or discomfort and altered bowel habits (diarrhea, constipation, or both).^62^, ^63^ This is another example where clinicians have the flexibility to subclassify a condition further when using the R U MOVVING SOMe classification system. The subclassification of IBS can be guided by the ROME criteria, a standardized, symptom‐based diagnostic criterion that has evolved through multiple iterations (currently ROME IV).^62^, ^64^, ^65^ These criteria classify IBS into subtypes: IBS with constipation (IBS‐C), IBS with diarrhea (IBS‐D), and IBS with mixed bowel habits (IBS‐M).^65^ Notably, IBS is part of a group of functional gastrointestinal disorders called disorders of gut‐brain interaction (DGBI).^64^, ^66^, ^67^ These conditions are characterized by altered bidirectional communication between the gut and the CNS, specifically the brain. In DGBI, disturbances in this interaction lead to various gastrointestinal symptoms, including abdominal pain, bloating, and altered bowel habits, without identifiable structural or biochemical abnormalities in the gut.^65^, ^66^ The underlying pathophysiology is multifactorial, encompassing altered gastrointestinal motility, visceral hypersensitivity, alterations in mucosal and immune function of the gut, changes in microbiota, CNS processing abnormalities, and psychological factors. In addition to IBS, other DGBI disorders include functional dyspepsia, functional abdominal bloating, and distention.^66^

If lower abdominopelvic pain is present, this category may also include inflammatory bowel disease (IBD), which is separated into two major subtypes: Crohn's disease and ulcerative colitis. Although these conditions differ in histological features (location within the gastrointestinal tract, depth of inflammation, and histologic markers), they have some common pathophysiologic mechanisms.^62^, ^68^ These include a dysregulated immune system, the influence of environmental triggers, and a significant component of genetic susceptibility.^65^, ^68^

### 3.14 S – Sensitization/Nociplastic

This category describes a state of altered nociceptive processing in the CNS, resulting in heightened pain perception. This phenomenon encompasses both peripheral and central sensitization and contributes to what is now termed nociplastic pain.^69^, ^70^, ^71^ According to the International Association for the Study of Pain (IASP), nociplastic pain is defined as “pain that arises from altered nociception despite no clear evidence of actual or threatened tissue damage causing the activation of peripheral nociceptors or evidence for disease or lesion of the somatosensory nervous system causing the pain”.^72^ This highlights that the pain experience is driven by dysfunctional pain processing within the CNS system rather than ongoing peripheral input.^69^, ^70^, ^72^

Sensitization is a key mechanism underlying nociplastic pain that can occur at both central and peripheral levels. Peripheral sensitization is defined by the IASP as “increased responsiveness and reduced threshold of nociceptors in the periphery to the stimulation of their receptive fields”.^72^ This can be triggered by inflammatory mediators or nerve injury, leading to increased sensitivity to stimuli in the affected area. The IASP definition of central sensitization is “increased responsiveness of nociceptors in the central nervous system to their normal or subthreshold afferent input”.^69^, ^70^ This involves changes in synaptic efficacy and neuronal excitability within the spinal cord and brain. This results in amplified pain signals, expanded receptive fields, and prolonged pain perception even after the initial peripheral stimulus resolves.^70^, ^73^

Nociplastic pain, resulting from these sensitization processes, can manifest as a primary pain condition, where the altered central pain processing is the dominant driver of the pain experience and is independent or persistent even when peripheral pain generators are adequately treated.^70^ Alternatively, sensitization can also act to amplify and exacerbate pain originating from other peripheral sources, such as nociceptive pain (resulting from tissue damage or other peripheral conditions) or neuropathic pain (resulting from a lesion or disease of the somatosensory nervous system).^69^, ^70^, ^74^ Notably, nociplastic pain can be identified in a subset of patients across nearly all chronic pain conditions.^37^ It is thought to be the dominant mechanism in chronic overlapping pain conditions (see below).^37^, ^75^ The hallmark symptoms of nociplastic pain are widespread pain throughout the body, generalized sensory sensitivity (i.e. heightened sensitivity to both internal and external stimuli), fatigue, non‐restorative sleep, and cognitive dysfunction.^76^ Consequently, the Sensitization/Nociplastic category may be used as a primary categorization or in conjunction with chronic pain conditions that may be placed in other categories of R U MOVVING SOMe.

### 3.15 O – Overlapping pain conditions

The term chronic overlapping pain conditions (COPCs) is frequently used within the National Institutes of Health (NIH) and collaborating research communities to refer to the frequent co‐occurrence of multiple chronic pain conditions in a single individual.^37^ Disorders that are frequently observed to occur together include fibromyalgia, IBS, chronic fatigue syndrome/myalgic encephalomyelitis (CFS/ME), temporomandibular joint disorders (TMD), IC/BPS, vulvodynia, migraine and other headache disorders, chronic low back pain (cLBP), and endometriosis.^36^, ^37^ Notably, although some of these conditions are not pelvic in nature, the co‐existence of two or more distinct chronic pain conditions in the same person would qualify for classification in this category. Research indicates that these conditions share common mechanisms, including central sensitization, autonomic nervous system dysfunction, genetic predisposition, psychological distress, risk factors, and treatment challenges.^36^, ^37^, ^75^ Clinically, the presence of nociplastic pain symptoms and/or the co‐occurrence of multiple chronic pain conditions is associated with greater pain intensity, higher levels of disability and functional limitations, poorer quality of life, increased healthcare utilization, and decreased likelihood of responding to therapies aimed at peripheral mechanisms (e.g. surgery).^5^, ^75^

The differentiation between nociplastic pain and COPCs may erroneously imply that they are distinct processes. In fact, nociplastic pain functions as a common underlying mechanism for COPCs. It is essential to clarify that the classification's inclusion of both sensitization and COPCs is intended to reflect the clinical features of nociplastic pain, which can manifest independently of COPCs. This means that patients without COPCs can still exhibit clinical features of nociplastic pain. Nonetheless, the inclusion of COPCs underscores the frequent overlap of these conditions and the heightened lifetime risk of developing additional pain conditions when one is already present. The overlapping pain conditions category should be used with other relevant categories within the R U MOVVING SOMe classification system and it is essential to provide additional detailed documentation that specifies the individual chronic pain conditions thought to be contributing to the patient's pain. This documentation is crucial for comprehensively understanding the patient's pain profile and facilitating multimodal management strategies.

### 3.16 Me – Mental health

Mental health conditions exhibit a significant and complex interplay with CPP;^5^, ^21^, ^75^, ^77^, ^78^ as such, the stakeholders and SC determined that mental health conditions are an essential part of any classification system that describes CPP. Psychological factors, including anxiety, depression, and trauma‐related disorders, such as post‐traumatic stress disorder (PTSD), history of assault or abuse, and adverse childhood experiences, are frequently comorbid with CPP, suggesting a bidirectional relationship.^77^, ^78^, ^79^ Although the persistent and often debilitating nature of CPP can understandably contribute to the development or exacerbation of mental health challenges, pre‐existing or co‐occurring psychological states can also significantly influence the perception and experience of pelvic pain.^80^, ^81^ Specific cognitive states, such as rumination (repetitive and passive dwelling on negative thoughts and pain sensations) and magnifying (an exaggerated and negative appraisal of pain and its potential consequences, also known as “catastrophizing”), have been shown to amplify pain intensity, increase emotional distress, and contribute to the chronicity of CPP.^21^, ^73^, ^80^ These maladaptive cognitive patterns are associated with heightened attention to pain signals, increased physiological arousal, and impaired effective coping strategies, thereby perpetuating the pain cycle.^73^, ^77^ Furthermore, shared neurobiological pathways involving stress response systems, inflammatory processes, and altered pain modulation may underlie the observed association between mental health and CPP, underscoring the importance of addressing psychological well‐being in the comprehensive management of individuals with CPP.^75^, ^82^, ^83^ As previously mentioned, evaluating mental health factors is recommended across all conditions within this classification system.

## 4 DISCUSSION

The development of the R U MOVVING SOMe classification system is a major advancement in the field of CPP. This novel system achieved through broad consensus among diverse stakeholders—including medical experts, patient advocacy organizations, professional societies, researchers, and educators—underscores the unmet need for a pragmatic and comprehensive framework to categorize the multifactorial nature of CPP. The complexity of CPP conditions has often hindered effective communication, diagnosis, and the development of targeted treatments, highlighting the importance of this unified approach. Although some conditions categorized in R U MOVVING SOMe can involve acute pain flares, their classification within this system is characterized by failure to initial therapy, persistent duration, a lack of a definitive cure, and significant impairment of quality of life, encompassing physical, emotional, and social functioning.

At its core, the R U MOVVING SOMe system was designed primarily with clinical utility in mind, reflecting the often overlapping and intricate patient presentations encountered in real‐world practice. Prioritizing a descriptive framework over hierarchical classification, the system allows clinicians to document co‐existing conditions, even when causal relationships or primary etiologies are unclear. For instance, a patient with endometriosis can be classified as CPP‐R (endometriosis), while one with overlapping conditions like IBS, IC, and endometriosis may be categorized as CPP‐O (endometriosis, IBS, IC). If mental health factors are also present, the classification extends to CPP‐OMe (endometriosis, IBS, IC, anxiety). Acknowledging the reality of overlapping pain conditions is expected to enhance its acceptance and utility among healthcare professionals, educators, and researchers. In addition, this approach fosters a patient‐centered approach to pain management by enhancing communication between patients and clinicians and validating the complexity of the patient experience.

Several key features further enhance the utility of the R U MOVVING SOMe system. First, it offers flexibility in utilizing other accepted classification systems for more granular subclassification and allows for a multi‐layered approach to diagnosis. Second, because it allows for potential overlap where a condition might conceivably fit under multiple headings (e.g. vulvar herpetic neuralgia under both the “Vulvovaginal” and “Neurologic” categories), the system serves as a practical tool for clinicians, enabling them to focus more on identifying and documenting all potential causes of a patient's persistent pain, thereby facilitating thorough clinical consideration during its application. Lastly, R U MOVVING SOMe is envisioned as a dynamic classification system capable of evolving as our scientific understanding of CPP grows.

We will use a multifaceted approach to evaluate the system's performance and uptake, including regular literature searches to track publications referencing the R U MOVVING SOMe classification. In addition, we will increase accessibility and visibility by uploading the system to the IPPS and Pelvic Pain Education Program (PPEP) websites. We will utilize website analytics to monitor engagement on these platforms, specifically tracking the number of clicks and downloads related to the system's resources. Finally, we will actively track presentations at conferences and research projects that adopt or investigate the R U MOVVING SOMe framework to gauge its dissemination and impact within the scientific and clinical communities.

To further optimize and enhance its clinical application, efforts are already underway to provide additional guidance on symptom terminology to be used within the framework of this classification system, as well as its integration into a comprehensive biopsychosocial evaluation of CPP. Furthermore, we will continue to refine and expand the R U MOVVING SOMe system in response to ongoing scientific advancement related to CPP to improve health‐related outcomes for patients worldwide.

## AUTHOR CONTRIBUTIONS

All authors contributed to the Delphi design, reviewing and editing the manuscript content. GL and AW handled administrative work, survey dissemination, and data collection. Data cleanup and analysis were performed by MGM and GL. The final results were reviewed by JDVE, CA, SA, JC, SK, AWH, and MGM. The original manuscript was written by MGM and GL, while GL and AWH collected and analyzed all stakeholder comments. Subsequent drafts were prepared by MGM, GL, and AW, with editorial assistance from JDVE, CA, AWH, SA, JC, and SK. GL finalized the edits and submitted the completed manuscript. In addition, the project stakeholders contributed data, assisted with data interpretation, and edited earlier versions of the manuscript.

## CONFLICT OF INTEREST STATEMENT

The authors declare no direct or obvious conflicts of interest related to the content of this manuscript, and no one involved in the project received payment for the content or work that went into its preparation. The authors disclose their affiliations and financial interests as follows: MGM has received consulting fees from Sumitomo; JDVE has received payment for lectures from Johnson & Johnson and Gedeon Richter; CA has received payment from Pfizer for symposia and has participated on advisory boards for Pfizer and Abbvie; SA has received consulting fees from Sumitomo, Bayer, Organon, and Gesynta, lecture fees from Gedeon Richter, and author royalties from UpToDate; AWH has received grant funding from EU, UKRI, NIHR, CSO, Wellbeing of Women, and Roche Diagnostics, consultancy fees from Roche Diagnostics, Gesynta, and Theramex, lecture fees from Gedeon Richter and Theramex, serves as a Specialty Advisor to the Scottish Government's Chief Medical Officer for Obstetrics and Gynaecology, participates on the PANDA DMC Data Safety Monitoring Board or Advisory Board, and holds UK Patent 2 217 921·2; SK has received a grant for the Serene Trial, royalties from the Pelvic Surgical Atlas, and consulting fees from Johnson & Johnson and Smith & Nephew; GL has received a research grant from the US Department of Defense and consulting fees from SoLá Pelvic Therapy. All other authors have no conflicts of interest.

## Data Availability

The authors are committed to transparency and will share de‐identified data upon request. Interested parties should contact the corresponding author to facilitate access to the data.
